# Underlying reasons why some people haven’t tested for HIV – a discourse analysis of qualitative data from Cape Town, South Africa

**DOI:** 10.1080/17290376.2021.1977686

**Published:** 2021-09-13

**Authors:** Kyla Meyerson, Graeme Hoddinott, Tamryn Nicholson, Sue-Ann Meehan

**Affiliations:** Department of Paediatrics and Child Health, Faculty of Medicine and Health Sciences, Desmond Tutu TB Centre, Stellenbosch University, Cape Town, South Africa

**Keywords:** Barriers, HIV testing, men, never tested

## Abstract

Reported barriers to HIV testing over the last 15 years have remained consistent, despite improved service offerings. We aimed to probe deeper by exploring how people who have never tested construct HIV testing in their talk. We used this to suggest underlying psychosocial barriers to testing even when there is high availability. We enrolled 14 participants who reported that they had never tested for HIV and conducted individual, open-ended interviews. The data were organised thematically with theory-generative interpretations informed by discourse analysis. Reasons for not testing reported reflect similar barriers identified in previous research. Deeper probing identified three discursive processes by which participants explained why they had never tested for HIV, suggesting that the way participants used ‘reasons’ in their talk is an indicator that the participants were repeating ‘tropes’. While aware of HIV testing facilities, participants still chose not to test. Influences on the choice to test or not were positioned as outside of the person’s control. These findings suggest that there *are* deeper reasons why some people have not tested and that these will not be resolved through merely increasing accessibility to testing services. We recommend increased consideration of the psychosocial implications of testing in service delivery.

## Introduction

South Africa has the highest HIV disease burden in the world; in 2018, 7.7 million people were living with HIV, of whom 4.8 million (62%) were receiving ART (UNAIDS, 2020). HIV testing remains vital as an entry point to the HIV care and treatment cascade. South Africa has increased access to HIV testing over the last decade and currently more than 90% of people living with HIV know their status (UNAIDS, 2019). However, this total statistic hides unequal uptake by subgroups, for example substantially lower among men than women (Grobler, Cawood, Khanyile, Puren, & Kharsany, 2017; Johnson, Rehle, Jooste, & Bekker, 2015). It is critical to understand the barriers to testing in order to improve HIV test uptake, especially among these subgroups.

South Africa implemented a successful national HIV testing campaign in 2010/2011 to increase the availability and uptake of HIV testing services; 7.2 million individuals tested for the first time (Maughan-Brown, Lloyd, Bor, & Venkataramani, 2016). The provider-initiated strategy relied on health providers routinely offering HIV tests to all individuals attending health facilities, regardless of signs of HIV infection (South African National Department of Health, 2016). Community-based HIV testing strategies (including mobile and door-to-door testing) were implemented to reach populations not typically accessing health facilities (Geoffroy, Schell, Jere, & Khozomba, 2017; Meehan, Naidoo, Claassens, Lombard, & Beyers, 2014). Both strategies continue to be part of HIV testing services in South Africa, in addition to HIV self-testing, which became part of the National HIV Services policy in 2016 (Venter et al., 2017), in a further attempt to reach under tested populations.

Research conducted prior to this testing campaign identified the following barriers to testing: lack of knowledge about HIV and location of testing sites, low perceived risk of infection, no perceived physical symptoms, fear of testing positive, discrimination and stigma (Haile, Chambers, & Garrison, 2007; Hutchinson & Mahlalela, 2006; Peltzer, Matseke, Mzolo, & Majaja, 2009). Following policy changes and the implementation of provider-initiated and community-based testing, the barriers reported remained similar (Macphail, Pettifor, Coates, Rees, & Cantab, 2013; Strauss, Rhodes, & George, 2015; Weihs & Meyer-Weitz, 2016). The consistency of these findings, despite targeted interventions, indicates that previous research may not be tapping into the core reasons for why some people do not test for HIV.

A recent scoping review which included studies across sub-Saharan Africa listed the same key barriers to men not testing; knowledge of HIV, fear of testing positive for HIV, stigma associated with HIV, healthcare providers’ services, confidentiality, and clinic setting (Hlongwa, Mashamba-Thompson, Makhunga, & Hlongwana, 2020). Over time, men have consistently provided the same reasons for not testing. Male-oriented interventions have been developed and implemented to address some of the key reasons (Hlongwa, Mashamba-Thompson, Makhunga, & Hlongwana, 2019; Van Rooyen et al., 2019). While these have shown some success, key challenges remain as HIV testing uptake among men overall remains sub-optimal. Gaining insight into the underlying reasons people give for their non-testing is vitally important, especially amongst men.

In this exploratory study, we returned to an existing data set where our initial thematic analysis had suggested reasons for not testing were similar to those reported in the literature (and described above). We re-looked at how people who had never tested talked about HIV testing, how they justified not testing, and how they positioned themselves in relation to testing. We aimed to probe deeper and suggest underlying, and under reported, psychosocial barriers to testing in the context of the high availability of HIV testing services.

## Methods

### Setting

Data collection took place in five communities within the Cape Metro District, Western Cape Province of South Africa. These communities are densely populated and resource-scarce, dwellings range from formal to informal and unemployment, substance abuse and disease prevalence are high. Antenatal HIV prevalence ranged between 17.7% and 27.1% across these communities at the time that data was collected in 2015 (Department of Health Republic of South Africa, 2015).

HIV testing services (HTS) were available free of charge and overall were readily accessible either at public primary health care (PHC) facilities or through community-based modalities like stand-alone centres (fixed sites) and mobile services (using ‘pop-up’ tents and a mobile van).

### Design and sampling

A larger study enrolled 29 participants. Of these, 15 had tested for HIV at a community-based HIV testing service (five reported as first-time testers, ten as repeat testers) and 14 reported never having tested for HIV. This nested study includes only the participants who reported that they had never tested for HIV. These participants were recruited door-to-door from households in close proximity to the 15 participants who had tested at a community-based service. Of the participants who reported never having tested 11/14 (79%) were men aged 18–35 years.

### Data collection

Fourteen interviews were conducted between July and December 2015 in private spaces (participants’ homes, workspaces or parked cars). Graduate research assistants conducted interviews in participants’ chosen languages (Xhosa or English). They used an interview guide that included questions related to testing (e.g. Why have you never tested for HIV?) and questions aimed at eliciting narratives that showed their positioning relative to HIV (e.g. Please tell us of a time when you thought about going for an HIV test and then decided not to). Interviews lasted on average approximately 35 min. Each interview was digitally recorded and later transcribed verbatim by the interviewers, and interviews conducted in Xhosa were translated into English after being transcribed.

### Ethical considerations

To maintain confidentiality, participants were assigned pseudonyms prior to transcription and translation. The study was conducted according to the Declaration of Helsinki and approved by the Stellenbosch University Health Review Ethics Committee (Reference number: S12/02/059). No participants received any incentive or reimbursement for taking part in the study.

### Analysis

Data were organised thematically and analysed from a constructionist perspective. The analytic process followed steps outlined by Braun and Clarke (2006) and Terre Blanche, Kelly, and Durrheim (2006). This included a familiarisation period, whereby transcripts were read and reread. The first and third authors discussed coding of the transcripts, first with terms used by the participants and then terms more appropriate for the study. Similar concepts were grouped together to form themes. These were then checked against the data. The findings from this initial analysis were similar to those reported in the literature. However, we were also sceptical of the findings because of the many changes in reasons given by participants (see below). We, therefore, returned to the data and then used critical discourse analysis (Fairclough, Mulderrig, & Wodak, 2006; Potter & Wetherell, 2004; Van Dijk, 1993; Wetherell, Taylor, & Yates, 2001) to interrogate how HIV testing was constructed, positioned and used by participants. This type of analysis goes beyond the face value presentation of reasons by the participants. See (Antaki, Billig, & Potter, 2003) and (Hepburn & Potter, 2003) enabled the authors to suggest alternative explanations for why the participants had never tested consistent with theory generative research (Corley & Gioia, 2011; Timmermans & Tavory, 2012).

## Findings

Overall, we identified three discursive processes by which participants explained why they had never tested for HIV: ‘shifting obstacles’; ‘hyperbole about the effect of learning that one is living with HIV’; ‘testing as a result of external events that have not yet happened in their lives’. We present each with one detailed worked example below, but all were interwoven throughout the participants’ narratives about why they had not tested. We suggest that the commonality of these discursive processes is an indicator that the participants were repeating ‘tropes’ rather than more powerful underlying reasons for not testing (Hammersley, 2003; Shotter, 2014).

### Shifting obstacles

Participants justified not having tested by citing a lack of testing facilities, time or trust in the facilities themselves. These explanations resonate with those reported in much of the literature reporting thematic descriptions of qualitative data. However, when challenged even only gently by the researcher, participants shifted and offered an alternative explanation. For example, Xola, a 32-year-old man, begins by saying that he does not test because he doesn’t have a chance and facilities are scarce, but later changes to say he does not trust the testing services. Throughout the extract he also unnecessarily repeats and over-emphasises that he wants to test and does not have a problem testing:

*Extract One*

Xola: I don’t have a reason [to test] but I want to. I would like to test but, but no what happens is I don’t get a chance [to test] or testing facilities are scarce you see [do you understand]?

Researcher: mh so

Xola: [interrupting researcher] I don’t have a problem with testing.

Researcher: so you you [stuttering] don’t have like a specific reason [for not testing] other than being busy?

Xola: no, there’s no problem [with testing].

Researcher: like like [stuttering] do you see sometimes that maybe there are these mobile clinics or tents [in the community] that are are [stuttering] standing, the ones where people shout ‘go test for HIV or TB’ or?

Xola: yes [pause] no the other thing is I usually don’t trust these tents [mobile clinics] that are being assembled anywhere [in the community] you see? [do you understand] then go [to go] and draw bloods [from] those things. So, now I don’t trust those things I ignore them.

When asked why he was not tested, Xola begins to recite reasons for not having tested. He then asks, ‘do you understand?’ in an attempt to elicit the researcher’s agreement. However, contrary to what Xola seems to expect, the researcher produces a non-comitial response. Xola interrupts immediately defending against the idea that the researcher might assume that his lack of testing could in fact indicate a problem. This interaction may indicate that Xola is trying to justify that his reasons for not testing are external and not within him.

He goes on to provide another explanation, replacing the lack of facilities with a lack of trust in the facilities that he does in fact have access to but ‘ignore[s]’. This tells us that the lack of facilities is unlikely to be at the root of Xola’s not testing and must be supplemented by another reason to be more convincing. This elaboration is again questioned by the researcher:

*Extract Two*

Researcher: so you don’t trust that maybe the the [stuttering] equipment that they use [in the tents] is it maybe [in] accurate or what?

Xola: [clicks with his mouth] and another thing you go and say you are going for a test and then these people [community members] then judge you and look at you with the wrong eye [with a judgemental look], [and] these other ones [community members] [participant laughs] you see [do you understand]?

Researcher: so you are scared [of] the reaction, people’s reactions from the community?

Xola: yes, it’s like that

Researcher: oooh okay from your friends, what will they say?

Xola: mh

Researcher: okay, so if maybe you are afraid of the public or the community or friends, have you ever considered that okay maybe I should go to to [stutters] a private doctor and get tested there instead? [breathes in] so that no one will know when you are in the consultation room, what you are there for [the reason for the consultation]?

Xola: yes it’s like that

Researcher: so have you ever considered maybe thinking of going to a private doctor for HIV testing?

Xola: yes, I’m interested to go there but I don’t have time

Researcher: [clicks with his mouth] it’s [the problem is] the time?

Xola: mh

The researcher questions the source of Xola’s lack of trust and Xola clicks his mouth before responding by shifting his focus from misgivings about testing facilities to fear of judgement by those who know about his potential testing. These clicks perform a hedging function. By producing small barriers within the talk, Xola is indicating that the conversation is not running very fluidly, perhaps flagging instances where he is reproducing reasons for not testing which might not serve up to the researcher’s standards of a ‘legitimate’ barrier to HIV testing. Finally, the researcher proposes a private doctor as a solution to the problem of community stigma and lack of trust. To this final challenge, Xola states that he doesn’t ‘have time’.

What are we to make of this exchange? On the one hand, Xola has reproduced many of the reasons provided in previous research for not testing (lack of facilities, lack of trust in the facilities, fear of community judgement and a lack of time). Perhaps there are just many reasons why Xola does not test. Alternatively, when the researcher provides a solution, the participant introduces another barrier. Perhaps there always being another reason for not testing presented without much thought, suggests that our participant’s primary reason for not testing is not addressed in the content of this response. Rather, plausible reasons are presented, and when challenged, replaced with others. It appears that contrary to Xola’s initial statement, he does have a ‘problem with testing’ but that this problem is something that he cannot say or does not know.

### Hyperbole of the effect of receiving a positive test result

Many participants positioned the psychological distress resulting from a positive diagnosis as something that will kill them before the disease does, thereby exaggerating the potential effect of an HIV positive diagnosis. For example, Sibusiso a 23-year-old man constructs *learning* that one is living with HIV as worse than HIV itself:

*Extract Three*

Sibusiso: mm my main reason for me, it’s like I don’t know whether it’s fear of the unknown or I don’t know if I fear that thing [HIV], you see [do you understand]? It’s as if I fear that thing [HIV]. If I can go the hospital and get there to test [for HIV] and then let’s say they discover that I’m HIV positive, by then psychologically I will be affected. I don’t know how to explain it but I, the way I see it, it’s as if it’s gonna affect me more in terms of thinking [about HIV] [be]cause I will not be free like [I] will already know that I live with the virus [HIV] you see [do you understand], that thing? That’s my main reason why I do not want to go and test.

Sibusiso does not construct an HIV test as an opportunity to learn his status, rather he constructs testing positive for HIV as an immediate death sentence. Knowing his status rather than living with HIV, in this instance, is spoken about as being psychologically traumatising and as something that would prevent him from freely living his life. Sibusiso’s not testing is positioned as a safer way to avoid distress and death. Therefore, exaggerating the consequences of testing justifies not testing as a rational decision and thereby preventing the individual from having to confront their underlying anxiety around testing for HIV. However, the consequence of not testing may mean that the individual will potentially remain in denial of the value of HIV testing in maintaining good health.

### Testing dependent on external events

Participants spoke of possible future testing through chance events that would typically happen to them rather than being initiated by them. For example, Luthando a 23-year-old man talked about his hope to test one day in the future:

*Extract Four*

Researcher: okay so do you think that maybe you would go and get tested in the future? Would you ever think that maybe ‘okay, now it’s time I should go and get tested’?

Luthando: no, I still do have that hope I don’t know where it comes from but I do have that thing that says, ‘yes, one day I would go and get tested, also but I don’t know when.’

By using the word ‘hope’, Luthando positions himself as waiting for a day where unspecified, predetermined events will lead to his testing for HIV. He continues ‘but I don’t know when’, constructing himself as uninformed of his own plan of action, indicating the extent to which testing falls outside of his control.

Additionally, participants typically positioned decisions to test as dependent on medical events, e.g. pregnancy, circumcision or emergency medical events where they perceived no choice but to test. For example, Mpho a 29-year-old man suggested that testing was necessitated by the emergence of HIV symptoms:

*Extract Five*

Mpho: The reasons why I haven’t tested yet is because I haven’t noticed anything happening to me yet [no physical symptoms that might indicate that the participant has HIV] uu nothing happening on my body. I mean I do think about going to test but I am lazy to test to go there because of work.

Mpho justifies not testing because he does not have HIV symptoms, saying ‘I haven’t noticed anything happening to me yet’. His framing of testing as motivated by the emergence of symptoms and his use of the word ‘yet’ makes it seem as though testing is needed but is not a process that can be set in motion by the decision alone. Mpho positions HIV testing as a process of confirmation rather than a check-up. He will only test when he ‘knows’ that he is HIV positive.

Additionally, the participants (most men) commonly constructed testing in terms of partner actions, thereby framing themselves as non-active in the HIV testing process. For example, Khaya a young man who did not specify his age talks about his partner testing:

*Extract Six*

Researcher: Okay do you think you will go to test for HIV in the long run [in the future]?

Khaya: No, I will go in the long run [in the future] but there are times when you see places where testing is carried out, even then you become reluctant to go and decide you are not ready yet. As I am saying, I will wait to see what is the situation [HIV status] with [of] my girlfriend. She must go first so that I can see what is going on [her HIV status].

Researcher: Okay, if your girlfriend goes are you going to do the same?

Khaya: If my girlfriend goes I will have no choice but to go too. By testing she would have forced me to go, you see [do you understand]? The thing is if I go to the clinic or the places that do testing, all I think about is that they will say I have it [HIV], do you understand? Yes, I don’t trust myself, it is as if they are going to say I have it [HIV] do you understand? That is why I do not go, it is like I won’t go out without them saying I have it [HIV].

Khaya initially references reluctance to test even when seeing ‘places where testing is carried out’. He attributes this to not being ready and suggests that he would rather wait until after his girlfriend has tested. He places testing outside of his control and displaces testing responsibility onto his partner. Concluding his account, he says ‘I won’t go out without them saying I have it’, demonstrating a paradoxical unwillingness to test unless he can be sure of the outcome of the test in advance.

From the above, it is clear that, while participants are able to imagine testing in the future, they are only able to do so with the assistance of external events. Luthando hopes that testing will happen to him rather than deciding to seek out testing. Khaya will only test if forced to by either his partner or his partner’s status. These participants do not engage with testing as something that can be undertaken and motivated by choice.

## Discussion

We explored the reasons why people choose not to test for HIV despite increased access to HIV testing services in our setting. The findings, considered from a purely content-based approach, are analogous to those outlined in previous research – lack of facilities, lack of trust in the facilities, fear of community judgement and a lack of time (MacPhail, Pettifor, Moyo, & Rees, 2009; Meehan, Draper, Burger, & Beyers, 2018; Musheke et al., 2013; Okal et al., 2020). Individuals do not choose to test mainly because services are not available with sufficient convenience.

Looking more closely at how participants talk about testing better enables us to see the complex role that testing plays in their lives. Participants were aware of HIV testing facilities yet chose not to test. We have demonstrated empirically that participants’ reasons for not testing reported in simple thematic analyses of qualitative data or indeed of survey data should be treated with caution – because they seem so open to change when challenged.

This finding highlights the importance of thinking about the manner in which HIV testing is presented within the health system to create demand for services among specific sub-groups (Treger & Tank, 2019) and to encourage better uptake by men specifically (Cornell, Cox, & Wilkinson, 2015). The underlying issues which prevent individuals from testing, may not be explicitly understood by these individuals, making them unable to articulate the ‘real’ reason for not testing. Previous studies have highlighted the perceived psychological burden of living with HIV (Musheke, Merten, & Bond, 2016), structural stigma (Bonnington et al., 2017), and gender-based norms that encourage men to participate in risk-taking behaviour (Gibbs, Willan, Misselhorn, & Mangoma, 2012; Shand, Thomson-de Boor, van den Berg, Peacock, & Pascoe, 2014) and view HIV testing as a threat to their masculinity (Katirayi et al., 2017)as reasons for not testing. In our study, men’s gendered self-identity seemed important to both their decision not to test and the reasons they reported. We suggest that this may be a fruitful point for further analysis in future studies.

There are ongoing calls for further explanations for why people do not test even in the context of high access. There has been a call to develop theory-informed interventions and evaluations to change gender norms (Colvin, 2019), to develop empirical-normative research (Knight, Small, & Shoveller, 2016) and to develop a socio-cultural responsive prevention strategy (De Jesus, Carrete, Maine, & Nalls, 2015). Our analysis is a first step toward formulating an alternative hypothesis.

A strength of the study is the use of a sophisticated, two-step analytic approach. The researchers make an effort to explore beyond the content provided by participants of the barriers for not testing for HIV in order to explore the underlying psychosocial mechanisms involved in not testing for HIV. The findings and extrapolation from these are also appropriately couched as theory-generative rather than declarative proofs. They are suggested as a potential alternative explanation that requires future empirical testing. Additionally, we used a non-convenience sample, which minimised potential sampling bias.

Limitations to extrapolation from the findings include the small sample size collected in one health district. A critical discourse analysis introduces uncertainty into interpreting the reasons at face value, therefore we do not claim any certainty at the ‘real’ reason why some people do not test for HIV. Rather, this analysis highlights the importance of further data collection and deeper analyses. Although consistent with the gender and age profile of people who do not test, the participants were also almost all young men, so the findings may not be transferable to women or older people who do not test.

## Conclusions

Even when services are available, not everyone chooses to test for HIV. When asked, the manner in which individuals who had not tested responded, showed that they are not explicitly aware of the underlying issues which have prevented them from testing, as they were unable to articulate the ‘real’ reason for not testing. Interventions to promote testing will need to extend to changing ideas around testing in a way that testing becomes a viable choice for those who have not tested. One suggestion is to address the underlying psychological processes of behaviour by promoting further talk around HIV testing decisions themselves (Barkway, 2013), thereby allowing potential testers to identify and engage with their reasoning for not testing and creating a non-judgemental environment in which testing concerns can be raised and, where possible, addressed; these approaches have waned in HIV testing services as ART has become more easily available, including the advent of treatment as prevention. This could also be operationalised as ‘community engagement’ sessions with targeted groups by creating ‘safe spaces’ in which to have these conversations. Instead of using counselling, as a pre- and post-HIV test measure to assist those who have already decided to test, conversation about testing with someone trained to also test for HIV could be a beneficial strategy. Other interventions could also focus more on the psycho-emotional aspects of pre- and post-HIV test counselling.

## References

[CIT0001] Antaki, C., Billig, M., & Potter, J. (2003). Discourse analysis means doing analysis: A critique of six analytic shortcomings. *Athenea Digital. Revista de Pensamiento e Investigación Social*, *1*(3), 14. 10.5565/rev/athenea.64.

[CIT0002] Barkway, P. (2013). *Psychology for health professionals*. Melbourne: Elsevier.

[CIT0003] Bonnington, O., Wamoyi, J., Ddaaki, W., Bukenya, D., Ondenge, K., Skovdal, M., … Wringe, A. (2017). Changing forms of HIV-related stigma along the HIV care and treatment continuum in sub-Saharan Africa: A temporal analysis. *Sexually Transmitted Infections*, *93*, 1–6. 10.1136/sextrans-2016-052975. PMC5739847 28736394

[CIT0004] Braun, V., & Clarke, V. (2006). Using thematic analysis in psychology. *Qualitative Research in Psychology*, *3*(2), 77–101. 10.1191/1478088706qp0630a.

[CIT0005] Colvin, C. J. (2019). Strategies for engaging men in HIV services. *The Lancet HIV*, *6*(3), e191–e200. 10.1016/S2352-3018(19)30032-3. 30777726

[CIT0006] Corley, K. G., & Gioia, D. A. (2011). Building theory about theory: What constitutes a theoretical contribution? *Academy of Management Review*, *36*(1), 12–32. 10.5465/AMR.2011.55662499.

[CIT0007] Cornell, M., Cox, V., & Wilkinson, L. (2015). Public health blindness towards men in HIV programmes in Africa. *Tropical Medicine and International Health*, *20*(12), 1634–1635. 10.1111/tmi.12593. 26325025

[CIT0008] De Jesus, M., Carrete, C., Maine, C., & Nalls, P. (2015). ‘Getting tested is almost like going to the Salem witch trials’: Discordant discourses between Western public health messages and sociocultural expectations surrounding HIV testing among East African immigrant women. *AIDS Care – Psychological and Socio-Medical Aspects of AIDS/HIV*, *27*(5), 604–611. 10.1080/09540121.2014.1002827. PMC4607024 25616443

[CIT0009] Department of Health Republic of South Africa. (2015). *Western Cape antenatal survey report – 2015*.

[CIT0010] Fairclough, N., Mulderrig, J., & Wodak, R. (2006). Critical Discourse analysis. In T. A. Van Dijk (Ed.), *Discourse studies – a multi-disciplinary introduction* (2nd ed., pp. 357–378). London: Sage Publications.

[CIT0011] Geoffroy, E., Schell, E., Jere, J., & Khozomba, N. (2017). Going door-to-door to reach men and young people with HIV testing services to achieve the 90–90–90 treatment targets. *Public Health in Action*, *7*(2), 95–99. 10.5588/pha.16.0121 PMC5493110 28695081

[CIT0012] Gibbs, A., Willan, S., Misselhorn, A., & Mangoma, J. (2012). Combined structural interventions for gender equality and livelihood security: A critical review of the evidence from southern and Eastern Africa and the implications for young people. *Journal of the International AIDS Society (JIAS)*, *15*(Suppl 1), 17362. 10.7448/IAS.15.3.17362 PMC3499786 22713350

[CIT0013] Grobler, A., Cawood, C., Khanyile, D., Puren, A., & Kharsany, A. B. M. (2017). Progress of UNAIDS 90-90-90 targets in a district in KwaZulu-Natal, South Africa, with high HIV burden, in the HIPSS study: A household-based complex multilevel community survey. *The Lancet HIV*, *3018*(17), 1–9. 10.1016/S2352-3018(17)30122-4. 28779855

[CIT0014] Haile, B. J., Chambers, J. W., & Garrison, J. L. (2007). Correlates of HIV knowledge and testing: Results of a 2003 South African survey. *Journal of Black Studies*, *38*(2), 194–208. 10.2307/40034975.

[CIT0015] Hammersley, M. (2003). Conversation analysis and discourse analysis: Methods or paradigms? *Discourse & Society*, *14*(6), 751–781. 10.1177/09579265030146004.

[CIT0016] Hepburn, A., & Potter, J. (2003). Discourse analytic practice. In C. Seale, G. Gobo, J. Gubrium, & D. Silverman (Eds.), *Qualitative research practice* (pp. 180–196). London: SAGE Publications.

[CIT0017] Hlongwa, M., Mashamba-Thompson, T., Makhunga, S., & Hlongwana, K. (2019). Mapping evidence of intervention strategies to improving men’s uptake to HIV testing services in sub-Saharan Africa: A systematic scoping review. *BMC Infectious Diseases*, *19*(1). 10.1186/s12879-019-4124-y. PMC6554953 31170921

[CIT0018] Hlongwa, M., Mashamba-Thompson, T., Makhunga, S., & Hlongwana, K. (2020). Barriers to HIV testing uptake among men in sub-Saharan Africa: A scoping review. *African Journal of AIDS Research*, *19*(1), 13–23. 10.2989/16085906.2020.1725071. 32174231

[CIT0019] Hutchinson, P. L., & Mahlalela, X. (2006). Utilization of voluntary counseling and testing services in the Eastern Cape, South Africa. *AIDS Care*, *18*(5), 446–455. 10.1080/09540120500213511. 16777636

[CIT0020] Johnson, L. F., Rehle, T. M., Jooste, S., & Bekker, L.-G. (2015). Rates of HIV testing and diagnosis in South Africa: Successes and challenges. *AIDS (London, England)*, *29*(11), 1401–1409. 10.1097/QAD.0000000000000721. 26091299

[CIT0021] Katirayi, L., Chadambuka, A., Muchedzi, A., Ahimbisibwe, A., Musarandega, R., Woelk, G., … Moland, K. M. (2017). Echoes of old HIV paradigms: Reassessing the problem of engaging men in HIV testing and treatment through women’s perspectives. *Reproductive Health*, *14*(1), 1–13. 10.1186/s12978-017-0387-1. 28982365 PMC5629810

[CIT0022] Knight, R., Small, W., & Shoveller, J. (2016). How do ‘public’ values influence individual health behaviour? An empirical-normative analysis of young men’s discourse regarding HIV testing practices. *Public Health Ethics*, *9*(3), 264–275. 10.1093/phe/phv031. 27790291 PMC5081038

[CIT0023] Macphail, C., Pettifor, A., Coates, T., Rees, H., & Cantab, M. (2013). You must do the test to know your status: Attitudes to HIV voluntary counseling and testing for adolescents among South African youth and parents. *Health Education and Behavior*, *35*, 87–104. 10.1177/1090198106286442. 16870815

[CIT0024] MacPhail, C., Pettifor, A., Moyo, W., & Rees, H. (2009). Factors associated with HIV testing among sexually active South African youth aged 15–24 years. *AIDS Care*, *21*(4), 456–467. 10.1080/09540120802282586. 19401866

[CIT0025] Maughan-Brown, B., Lloyd, N., Bor, J., & Venkataramani, A. S. (2016). Changes in self-reported HIV testing during South Africa’s 2010/2011 national testing campaign: Gains and shortfalls. *Journal of the International AIDS Society*, *19*(1), 1–10. 10.7448/IAS.19.1.20658. PMC4829657 27072532

[CIT0026] Meehan, S., Draper, H. R., Burger, R., & Beyers, N. (2018). What drives ‘first-time testers’ to test for HIV at community-based HIV testing services? *Public Health in Action*, *7*(4), 304–306. 10.5588/pha.17.0064. PMC5753785 29584797

[CIT0027] Meehan, S., Naidoo, P., Claassens, M. M., Lombard, C., & Beyers, N. (2014). Characteristics of clients who access mobile compared to clinic HIV counselling and testing services: A matched study from Cape Town, South Africa. *BMC Health Services Research*, *14*(1), 1–8. 10.1186/s12913-014-0658-2. 25526815 PMC4280046

[CIT0028] Musheke, M., Merten, S., & Bond, V. (2016). Why do marital partners of people living with HIV not test for HIV? A qualitative study in Lusaka, Zambia. *BMC Public Health*, *16*(1), 1–13. 10.1186/s12889-016-3396-z. 27561332 PMC5000425

[CIT0029] Musheke, M., Ntalasha, H., Gari, S., McKenzie, O., Bond, V., Martin-Hilber, A., & Merten, S. (2013). A systematic review of qualitative findings on factors enabling and deterring uptake of HIV testing in sub-Saharan Africa. *BMC Public Health*, *13*(1), 220. 10.1186/1471-2458-13-220. 23497196 PMC3610106

[CIT0030] Okal, J., Lango, D., Matheka, J., Obare, F., Ngunu-Gituathi, C., Mugambi, M., & Sarna, A. (2020). ‘It is always better for a man to know his HIV status’ – a qualitative study exploring the context, barriers and facilitators of HIV testing among men in Nairobi, Kenya. *PLoS ONE*, *15*(4), 1–22. 10.1371/journal.pone.0231645. PMC7159816 32294124

[CIT0031] Peltzer, K., Matseke, G., Mzolo, T., & Majaja, M. (2009). Determinants of knowledge of HIV status in South Africa: Results from a population-based HIV survey. *BMC Public Health*, *9*, 174. 10.1186/1471-2458-9-174. 19500373 PMC2700104

[CIT0032] Potter, J., & Wetherell, M. (2004). Discourse analysis. In M. A. Hardy & A. Bryman (Eds.), *Handbook of data analysis* (pp. 607–624). London: Sage Publications. 10.4135/9781446221792.n6.

[CIT0033] Shand, T., Thomson-de Boor, H., van den Berg, W., Peacock, D., & Pascoe, L. (2014). The HIV blind spot: Men and HIV testing, treatment and care in sub-Saharan Africa. *AIDS Bulletin*, *45*(1), 53–60. 10.1111/1759-5436.12068.

[CIT0034] Shotter, J. (2014). Rhetoric and argumentation. In Charles Antaki & Susan Condor (Eds.), *Rhetoric, ideology and social psychology – essays in honour of Michael Billig* (pp. 43–56). London: Routledge. 10.4324/9780203386088.

[CIT0035] South African National Department of Health. (2016). *National HIV Testing Services Policy 2016*. http://www.hst.org.za/publications/national-hiv-testing-services-policy.

[CIT0036] Strauss, M., Rhodes, B., & George, G. (2015). A qualitative analysis of the barriers and facilitators of HIV counselling and testing perceived by adolescents in South Africa. *BMC Health Services Research*, *15*(1), 250. 10.1186/s12913-015-0922-0. 26123133 PMC4484707

[CIT0037] Terre Blanche, M., Kelly, K., & Durrheim, K. (2006). Why qualitative research. In D. P. M. T. Blanche & K. Durrheim (Eds.), *Research in practice: Applied Methods for the social sciences* (pp. 269–284). Johannesburg: Juta and Company Ltd.

[CIT0038] Timmermans, S., & Tavory, I. (2012). Theory construction in qualitative research: From grounded theory to abductive analysis. *Sociological Theory*, *30*(3), 167–186.

[CIT0039] Treger, L., & Tank, S. (2019). *Landscape analysis: Men’s health programmes in South Africa* (Issue October).

[CIT0040] UNAIDS. (2019). Global AIDS update. In *Communities at the centre*. https://www.unaids.org/sites/default/files/media_asset/2019-global-AIDS-update_en.pdf.

[CIT0041] UNAIDS (2020). *Aids information*. 2020. Retrieved May 11, 2020, from https://aidsinfo.unaids.org

[CIT0042] Van Dijk, T. A. (1993). Principles of critical discourse analysis. *Discourse & Society*, *4*(2), 249–283. 10.1177/0957926593004002006o.

[CIT0043] Van Rooyen, H., Makusha, T., Joseph, P., Ngubane, T., Kulich, M., Sweat, M., & Coates, T. (2019). *Zwakala Ndoda*: A cluster and individually randomized trial aimed at improving testing, linkage, and adherence to treatment for hard-to reach men in KwaZulu-Natal, South Africa. *Trials*, *20*(1), 1–14. 10.1186/s13063-019-3908-0. 31888701 PMC6937627

[CIT0044] Venter, F., Majam, M., Jankelowitz, L., Adams, S., Moorhouse, M., Carmona, S., … Gray, A. (2017). South African HIV self-testing policy and guidance considerations. *Southern African Journal of HIV Medicine*, *18*(1), 9 pages. 10.4102/sajhivmed.v18i1.775. PMC5842980 29568643

[CIT0045] Weihs, M., & Meyer-Weitz, A. (2016). Barriers to workplace HIV testing in South Africa: A systematic review of the literature. *AIDS Care*, *28*(4), 495–499. 10.1080/09540121.2015.1109586. 26560013

[CIT0046] Wetherell, M., Taylor, S., & Yates, S. J. (Eds.). (2001). *Discourse as data – a guide for analysis*. London: Sage Publications.

